# On the Employment of Cupping-Glasses to the Spine, in Intermittent Fever

**Published:** 1849-12

**Authors:** 


*On the Employment of Cupping-Glasses to the Spine, in Intermittent Fever.*—M. Gondret submits the following method, which he states has never, in his hands, failed to cure intermittent fever: Apply eight or ten middle-sized cupping-glasses on each side the spinal column, from the neck downwards, and let them remain on for about thirty or forty minutes, without scarification, or, in other words, dry cupping. The time for applying the cups is the beginning of the cold stage, or, if it is possible, a short time before its accession, say a quarter of an hour; and this not only prevents the attack, but, at the same time, the hot fit and the sweating. In most cases, one application of the cups is sufficient to cure entirely an intermittent fever—however, in cases of long standing, it only modifies the time of the attack, and requires, for a complete cure, to be repeated three or four times.

In my own private practice, for these last twenty-seven years, I have never once met with a case of intermittent fever which has not given way to this simple treatment. The vacuum produced along the vertebral column, operates, I think, as a salutary derivative, which is easily understood, by considering the different anastomoses which exist between the vertebral arteries and those of the cerebral, such as the vertebral arteries, the circle of Willis, the bronchial, the cardiac, the esophagian, &c. I cannot affirm that this mode of treatment would succeed in your climate: at all events, the experiment can easily be made.

As to the seat of the disease, it is impossible to affirm, with absolute certainty. However, from my own observation, I am inclined to think that it is sometimes located in one organ, sometimes in another, and the treatment is based on that belief; thus, when there are headache, giddiness, heat, and heaviness of the head, I apply cups to the back of the neck, and sometimes take away one ounce or an ounce and a half of blood, which immediately relieves; if there is cough, difficulty of breathing, palpitation, &c., I apply them between the shoulders; and draw two or three ounces of blood, and so on. By following this plan, I always find the symptoms disappear in a short time.—*Encyclograp. Medicale.*

